# How patient stories can improve health services around the world

**DOI:** 10.1111/hex.12987

**Published:** 2019-10-21

**Authors:** Louise Condon

**Affiliations:** ^1^ Swansea University Swansea UK

1

A health board in Wales, UK^1^ holds ‘listening events’ where patients tell their stories of service use to NHS and local authority workers, and third sector organisations. Initially a few health professionals were reluctant to engage, saying that they knew their patients well and were familiar with their stories. After the first event one such health professional informed the organiser that, contrary to his expectations, he had learnt much from talking to his allocated patient and appreciated more of what service is like from the ‘other side’. This edition of HEX contains a number of papers which look at expectations and experiences of health services from the perspective of service users, their carers and health service providers.

The position of patients who cannot leave hospital because no care setting is available (irreverently called ‘bed blockers’ in the media despite objections from the head of the Royal College of Nursing as early as 2000^2^) is frequently viewed from the organisational side only. Everall et al conducted a scoping review that identified that patients and caregivers in this situation are distressed by the uncertainty, the mental and physical deterioration precipitated by prolonged hospital stay and lack of engagement in decision making. In order to convey the stories of frail, older people to primary care teams Grol et al conducted ‘mirror meetings’ whereby groups of patients inform service providers of their experience of care, under the guidance of an independent moderator.

Sometimes the views of patients and service providers differ, as in Han et al's study of decision‐making amongst older people with end stage renal disease. Autonomy is the right of patients to make decisions about their care without their healthcare provider trying to influence the decision^3^ and in Han's study some patients felt persuaded by doctors and family members to commence dialysis. Both patients and caregivers reported subsequent concerns about the financial and caregiving burden. Damman et al looked at the use of patient‐reported outcome measures (PROMS) in the decision making of patients with Parkinson's disease. While both patients and professionals were positive about PROMS, they valued them for different reasons, and both groups thought the other should introduce the subject. Autonomy was also an issue for adolescent boys in disadvantaged neighbourhoods in the Netherlands, who wanted to eat junk food as an important part of their social lives (Lems et al).

Making decisions about healthcare often involves considering risk. Pacific people in New Zealand are at higher risk of diabetes, and Schmidt‐Busby et al found misunderstanding of health risks in interactions with health providers. One participant described their devastation at having an incurable illness that might have been prevented at an earlier stage, which highlights the need to understand how better to communicate with patients. In a multi‐method study Hogden et al explored how risk is communicated and perceived, and concluded that effective risk communication needs to be finely tuned and timed to individual patient's priorities and information requirements. Two studies look at patients’ experiences after treatment (Samobrec et al and Blum‐Barnett et al). They found that the impacts of injury and survivorship are long‐lasting, affecting finances and recreational activities, in tandem with physical and emotional health. Schofield et al also stress the importance of the community context of illness experience.

Finally, a number of papers explore how patients can be involved in shaping the health services they use. In a Norwegian study Kvael et al looked at healthcare professionals’ experiences of patient participation in intermediate care, calling for greater emphasis on individualised rehabilitation and a recognition that psycho‐social aspects are crucial for patient participation. When older people move from hospital to home (Murray et al) their involvement with the process varies in accordance with interaction with health professionals as they attempt to resolve wellbeing goals. In a realist literature review Bergerum et al sought to establish how patients can be involved in healthcare quality improvement, a widespread aim of policy. Taking a co‐creation approach Mortimer et al worked with older women in Australia to generate interventions aimed at addressing blockages and service gaps in treatment pathways. Harris et al identify that, while patient and public involvement is an international requirement in diabetes research, little is known about how involvement relates to health outcomes; their study showed that understanding the community context and making trusting relationships were key in designing feasible and locally relevant research. A novel paper by Johnston et al explored how the public use reports on primary care performance, concluding that in this Canadian setting the primary purpose was to promote trust.

Involving the public is essential in all aspects of healthcare practice and research. This edition of HEX is a rich source of patients’ stories about their experiences of health services, which serve to inform and educate service providers, and to influence the structure of health services around the world.
